# Validation of whole-blood transcriptome signature during microdose recombinant human erythropoietin (rHuEpo) administration

**DOI:** 10.1186/s12864-017-4191-7

**Published:** 2017-11-14

**Authors:** Guan Wang, Jérôme Durussel, Jonathan Shurlock, Martin Mooses, Noriyuki Fuku, Georgie Bruinvels, Charles Pedlar, Richard Burden, Andrew Murray, Brendan Yee, Anne Keenan, John D. McClure, Pierre-Edouard Sottas, Yannis P. Pitsiladis

**Affiliations:** 1 Centre of Sports Medicine for Anti-Doping Research, University of Brighton, Eastbourne, UK; 2 Institute of Cardiovascular and Medical Sciences, College of Medical, Veterinary and Life Sciences, University of Glasgow, Glasgow, UK; 3 Brighton and Sussex Medical School, Brighton, UK; 4 Faculty of Medicine, University of Tartu, Tartu, Estonia; 5 Graduate School of Health and Sports Science, Juntendo University, Chiba, Japan; 6 School of Sport, Health and Applied Science, St Mary’s University, Twickenham, London, UK; 7 Centre for Sports and Exercise, University of Edinburgh, Edinburgh, UK; 8 Affymetrix, Santa Clara, CA USA; 9 BioKaizen Lab SA, Monthey, Switzerland; 10 Department of Movement, Human and Health Sciences, University of Rome “Foro Italico”, Rome, Italy

**Keywords:** Recombinant human erythropoietin, Whole blood, Transcriptome, Altitude, Exercise, Athlete biological passport

## Abstract

**Background:**

Recombinant human erythropoietin (rHuEpo) can improve human performance and is therefore frequently abused by athletes. As a result, the World Anti-Doping Agency (WADA) introduced the Athlete Biological Passport (ABP) as an indirect method to detect blood doping. Despite this progress, challenges remain to detect blood manipulations such as the use of microdoses of rHuEpo.

**Methods:**

Forty-five whole-blood transcriptional markers of rHuEpo previously derived from a high-dose rHuEpo administration trial were used to assess whether microdoses of rHuEpo could be detected in 14 trained subjects and whether these markers may be confounded by exercise (*n* = 14 trained subjects) and altitude training (*n* = 21 elite runners and *n* = 4 elite rowers, respectively). Differential gene expression analysis was carried out following normalisation and significance declared following application of a 5% false discovery rate (FDR) and a 1.5 fold-change. Adaptive model analysis was also applied to incorporate these markers for the detection of rHuEpo.

**Results:**

*ALAS2*, *BCL2L1*, *DCAF12*, *EPB42*, *GMPR*, *SELENBP1*, *SLC4A1*, *TMOD1* and *TRIM58* were differentially expressed during and throughout the post phase of microdose rHuEpo administration. The *CD247* and *TRIM58* genes were significantly up- and down-regulated, respectively, immediately following exercise when compared with the baseline both before and after rHuEpo/placebo. No significant gene expression changes were found 30 min after exercise in either rHuEpo or placebo groups. *ALAS2*, *BCL2L1*, *DCAF12*, *SLC4A1*, *TMOD1* and *TRIM58* tended to be significantly expressed in the elite runners ten days after arriving at altitude and one week after returning from altitude (FDR > 0.059, fold-change varying from 1.39 to 1.63). Following application of the adaptive model, 15 genes showed a high sensitivity (≥ 93%) and specificity (≥ 71%), with *BCL2L1* and *CSDA* having the highest sensitivity (93%) and specificity (93%).

**Conclusions:**

Current results provide further evidence that transcriptional biomarkers can strengthen the ABP approach by significantly prolonging the detection window and improving the sensitivity and specificity of blood doping detection. Further studies are required to confirm, and if necessary, integrate the confounding effects of altitude training on blood doping.

**Electronic supplementary material:**

The online version of this article (10.1186/s12864-017-4191-7) contains supplementary material, which is available to authorized users.

## Background

The performance-enhancing drug recombinant human erythropoietin (rHuEpo) stimulates red blood cell production and although the World Anti-Doping Agency (WADA) prohibits its use, is frequently abused by athletes. The early anti-doping approach was to set an upper limit for haemoglobin and haematocrit levels in an attempt to discover rHuEpo abuse [1]. The first direct analytical procedure to detect rHuEpo was introduced in 2000 and exploited the differences in the charge profiling of endogenously and exogenously produced Epo in urine by isoeletric focusing (IEF) [2]. The main limitations of this direct approach are a variable and short detection window and low sensitivity [3]. There is now an improved direct analytical test to detect rHuEpo using sarcosyl polyacrylamide gel electrophoresis (SAR-PAGE, a modified sodium dodecyl sulfate polyacrylamide gel electrophoresis) with increased discriminatory capacity compared to IEF and detection window of 24 to 85 h using blood and urine samples [4]. According to the WADA technical document (i.e. TD2014EPO), SAR-PAGE is currently recommended for rHuEpo detection in both the initial and confirmation testing procedures [5]. Despite these advances, important limitations in detection of the direct approach have prompted a paradigm shift to the indirect identification of the effect of the prohibited method and/or substance.

One of the initial indirect methods involved the use of altered haematological markers such as reticulocyte haematocrit, haematocrit and percent macrocytes for the detection of current and recent rHuEpo intake using the ON- and OFF-statistical models, respectively [6]. This further advance culminated in the development of the Athlete Biological Passport (ABP) [1], introduced in 2009 by WADA. The ABP monitors changes in the blood matrix of an individual over time using Bayesian inference techniques to establish an individual’s haematological profile that can reveal evidence of doping, not confined only to rHuEpo but also other forms of blood manipulation [7]. The stability of the ABP haematological parameters is limited to 48 h for reticulocytes and 72 h for haemoglobin from blood collection to analysis when samples are handled at 4 °C and a limit of 36 h has been recommended by WADA for improved analytical quality [8]. A particular advantage of the ABP approach is the incorporation of other evidence of doping, such as longitudinal performance data, additional biomarkers yet to be discovered and validated and other nonanalytical evidence [1]. Despite this significant advance, the detection of rHuEpo and blood doping in general, using the current ABP approach, remains unsatisfactory [9, 10]. For example, the application of the ABP failed to reveal any evidence of rHuEpo microdosing (i.e. 20–30 IU∙kg^−1^ body mass rHuEpo twice weekly for 8 weeks) in 10 healthy male subjects [11]. In addition, the haematological parameters of the ABP may be confounded with factors such as altitude exposure, since hypoxia may affect the blood variables and vascular volumes [12, 13]. Notwithstanding these limitations, the addition of other biomarkers to the ABP is envisaged to improve the sensitivity and specificity of the ABP model, and therefore substantially enhancing future doping detection strategies [1, 14, 15].

Current advances in omics technologies permit a global transcriptional, translational or epigenomic feature of a cell, tissue or organism under altered physiological or developmental conditions to be investigated. For example, a state-of-the-art omics approach has been successfully applied to epidemic diseases (e.g. [16, 17]) and cancer diagnosis [18]. Undoubtedly, the application of omics to the field of anti-doping will help reveal potential doping biomarkers. In a study by Varlet-Marie et al., the gene expression profile in response to darbepoetin alpha was determined using the serial analysis of gene expression (SAGE) by pooling whole-blood samples from 14 healthy, active subjects (50% male) into three SAGE libraries (before, during and after administration) [19]. The authors then confirmed the differential expression of 95 genes identified using SAGE in two well-trained male athletes by qPCR [19]. Five genes remained significantly expressed following a further high dose and microdose rHuEpo treatment in these athletes based on a fold change of 1.5 and a false discovery rate (FDR) of 0% [19]. This initial promise of improved discriminatory potential of the transcriptomic biomarkers to detecting doping, encouraged a number of other attempts to investigate the global gene expression patterns in whole blood or lymphocytes for the detection of testosterone, anabolic steroids, recombinant human growth hormone, gene doping, blood transfusions and rHuEpo (see review [14]). It should be noted that samples are often collected for anti-doping purposes at sporting or training venues after intense exercise [20] and prior intense exercise training has been reported to have significant impact on gene expression profiles of peripheral blood mononuclear cells [21] and white blood cells [22]. We previously carried out whole-blood gene expression profiling using a microarray-based approach to detect rHuEpo doping (i.e. 50 IU∙kg^−1^ body mass every two days for 4 weeks). Briefly, 34 of 45 selected genes from two independent groups following the microarray analysis were validated using a different quantification platform [23]. The limitations of this study were the absence of a control/placebo group and the now out-dated rHuEpo dosing regimen involving near clinical doses of rHuEpo as opposed to the more commonly used strategy involving rHuEpo microdosing [23]. With these limitations in mind, the aims of the present study were to investigate 1) whether the previously identified 45 transcriptional markers could detect rHuEpo microdosing in a randomised, double-blind, placebo-controlled cross-over study; and 2) whether these gene responses differ among rHuEpo microdosing and potential confounders such as strenuous exercise training and moderate altitude exposure.

## Methods

**Fig. 1:**
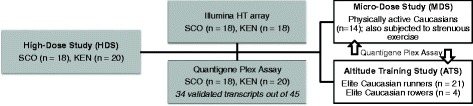
A simplified diagram of the study flow specifying sample size and analytical platforms used. SCO: Scottish cohort in the HDS. KEN: Kenyan cohort in the HDS

Forty-five candidate transcripts and five reference genes identified from the whole-blood transcriptome profiling in subjects administered with 50 IU∙kg^−1^ body mass of rHuEpo for 4 weeks (i.e. the rHuEpo high-dose study, see Additional file 1) [23] were interrogated in subjects participating in the rHuEpo microdose study and the rHuEpo confounders studies involving exercise and altitude training (Fig.1).

### Study design

#### Microdose study (MDS)

**Fig. 2:**
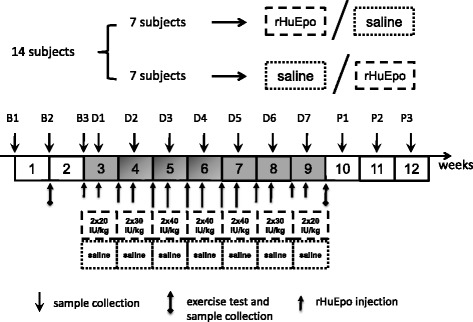
MDS experimental design. B: baseline; D: during rHuEpo administration; P: post rHuEpo administration

Fourteen endurance-trained healthy males (mean ± standard deviation (SD); age: 29.9 ± 4 yrs., height: 178.8 ± 4.5 cm, maximal aerobic capacity ($\overset{.}{\mathrm{V}}$O_2_max): 55.3 ± 4.7 ml∙kg^−1^∙min^−1^) at sea-level (Glasgow, Scotland) not involved in competition during the study period participated in a randomised, double-blind, placebo-controlled crossover microdose rHuEpo study. Written informed consents were obtained from all participants. The study was approved by the University of Glasgow Ethics Committee (Scotland, UK). The subjects received 20–40 IU∙kg^−1^ body mass subcutaneous injections of rHuEpo (NeoRecormon, Roche, Welwyn Garden City, UK) or equivalent saline (NaCl 0.9%, Baymed Healthcare Limited, Glasgow, UK; placebo injection) twice a week for 7 weeks (Fig. 2). All subjects received daily iron tablets providing approximately 105 mg of elemental iron derived from 350 mg of dried ferrous sulphate (Almus, Barnstaple, UK), while lactose (Minerals-Water, Purfleet, UK) substituted daily iron during the placebo trial.

All subjects were also subjected to a modified Wingate test comprising of 10 sprints of 10 s at baseline and during the week after the last rHuEpo or placebo injections (Fig. 2; performance data not included here). Specifically, after a 5-min cycling warm-up at 100 W, with a flat-out sprint at 3 min for 5 s, followed by a 5-min rest [24], each subject performed a series of ten, maximal effort 10 s sprints, separated by a 50 s rest interval. The subject then underwent 10 min of active recovery at 100 W followed by 110 min of rest.

#### Altitude training study (ATS)

Twenty-one elite endurance runners (12 males and 9 females) were recruited (mean ± SD; age: 23.2 ± 2 yrs., height: 175.3 ± 8.5 cm, body mass index (BMI): 19.7 ± 1.2 kg∙m^−2^). Informed, written consents were obtained from all participants. This study was approved by the Ethics Committee of the University of Brighton (England, UK). Participants were randomly assigned into an altitude group (*n* = 12; trained at Sierra Nevada, Spain, 2320 m, for approximately 2–3 weeks, with one participant returning from altitude after 8 days and another after 28 days) and a control group (*n* = 9; trained at sea level for approximately 2 weeks). Both groups followed the same training programme in preparation for a major international athletics competition.

Four elite male rowers were also recruited (mean ± SD; age: 25.3 ± 3.6 yrs., height: 193.3 ± 3.6 cm, BMI: 26.3 ± 1.2 kg∙m^−2^ and $\overset{.}{\mathrm{V}}$O_2_max: 63.8 ± 4.3 ml∙kg^−1^∙min^−1^). These athletes underwent 2-week of altitude training (Santa Caterina di Valfurva, Italy, 1850 m) in preparation for an international competition. Prior to and after the altitude exposure, these athletes remained in a hypoxic chamber/altitude room (16 m^2^; excluding training and for meals) set to an oxygen concentration equivalent to 2500 m for 5 days. Informed and written consent was obtained from all participants and approved by the Ethics Committee of the University of Glasgow (Scotland, UK).

### Blood sampling and RNA extraction

In the MDS, whole blood samples were collected in triplicate at baseline (approximately day −14, −7 and 0) and then once a week for 7 weeks during rHuEpo administration (approximately day 3, 10, 17, 24, 31, 38 and 45) and for 3 weeks post rHuEpo administration (approximately day 52, 59 and 66) (Fig. 2). Whole blood samples were also obtained before, immediately after the 10 sprints and 30 min after the last sprint following the modified Wingate test both at baseline and after the last rHuEpo or placebo injections (Fig. 2). In the ATS, whole blood samples were collected from the elite runners at baseline (approximately day −14), during (approximately day 10), 48-h-, 1-week-, 2-week- and 4-week-post altitude exposure, respectively. Samples were also obtained from the control group of elite runners at baseline (approximately day −14), 2-week- and 4-week-post the sea-level training period. Whole blood samples were collected from the elite rowers at baseline (day −14 and −10), during (day 5 and 22) and post (day 29, 33 and 39) simulated and natural altitude exposure (days are relative to the first day of the simulated altitude). For all studies, 3 mL whole blood sample was collected using the Tempus™ Blood RNA tube (Life Technologies, Carlsbad, CA, USA) and mixed vigorously with 6 mL stabilising reagent immediately after collection. The whole sample was incubated at room temperature for approximately 3 h and then stored at −20 °C before subsequent analysis. Three-millilitre whole blood was collected in K_3_EDTA tubes (Greiner Bio-One Ltd., Stonehouse, UK) for haematology analysis, and the whole blood was mixed thoroughly with the tube additive by gently inverting the tube 5–10 times as per the manufacturer’s instructions.

Total RNA was isolated from the whole blood collected using Tempus tubes according to the manufacturer’s instructions (Tempus™ Spin RNA Isolation Kit, Life Technologies, Carlsbad, CA, USA). The purified total RNA was eluted in 90 μL elution buffer and stored in three aliquots at −80 °C until further analysis. RNA quantity and purity was assessed by the Nanodrop^TM^ ND-2000 Spectrophotometer (Thermo Fisher Scientific, Wilmington, DE, USA).

### Haematological analysis

The mixed K_3_EDTA blood tubes (Greiner Bio-One Ltd., Stonehouse, UK) was tested on the Sysmex XT-2000i (Sysmex, Norderstedt, Germany) for the MDS and ATS elite rowers samples, or the Advia 2120i system (Siemens, Worldwide) for the ATS elite runners samples. Standard haematological parameters were measured (i.e. haemoglobin, HGB; haematocrit, HCT; and reticulocyte percent, RET%). These samples were measured in accordance with the WADA Athlete Biological Passport Operating Guidelines (version 4.0, 2013) [25]. The blood data was analysed using R lme4 (for applying the linear model) [26, 27] and phia (for post-hoc interaction analysis) [28] packages using a mixed design, with two within-subject variables (the rHuEpo/placebo trial and/or time covariates) as the fixed factors and subject as the random factor, in the MDS and ATS, respectively. Significance level was adjusted using the holm-bonferroni method.

### QuantiGene Plex experiment and data analysis

Two hundred nanogram RNA was run in duplicate for quantification of the 45 selected RNA targets and 5 reference genes (*ACTB*, *ACTR10*, *MRFAP1*, *PPIB* and *RAB11A*) in subjects participating in the MDS and ATS, using the QuantiGene Plex Assay (Affymetrix, Santa Clara, CA, USA). The resulting fluorescence signal was measured on the MAGPIX (Luminex, Austin, TX, USA). The median fluorescence intensity (MFI) values were viewed and exported from the xPonent software (Luminex, Austin, TX, USA) for statistical analysis. The MFI data was background subtracted and normalised to the geometric mean of the five reference genes. The coefficient of variation (CV) for assay precision was calculated using the duplicated samples. The “limma” function implemented in the R limma package [29] was used to perform the differential expression analysis based on the adjusted and log2 transformed data. Gene expression values were compared to the averaged baseline or the baseline in the MDS (i.e. in the rHuEpo and placebo groups, respectively), MDS exercise samples (i.e. post vs. pre exercise as well as before-after treatment comparisons in the rHuEpo and placebo groups, respectively) and ATS (i.e. in the altitude and control groups, respectively), where appropriate. Gene expression changes over time were also examined in rHuEpo vs. placebo in the MDS and MDS exercise samples and in altitude vs. control in the elite runners. Transcripts expression exceeding a FDR [30] < 0.05 and a 1.5 fold-change were considered meaningful in the current context.

### Adaptive model analysis of blood and molecular signatures

In the MDS, the adaptive Bayesian model was applied on the two primary markers (i.e. HGB and the stimulation index OFF-score) of the haematological module of the ABP. Parameters of the model, including within- and between-subject components of variance, were chosen to represent a modal population of Caucasian male athletes aged 20–40 yrs. [31]. The data were analysed by an investigator without knowing whether the profile was obtained from a rHuEpo or placebo sample. Following the unblinding, sensitivity was calculated based on the portion of samples in the rHuEpo group that produced at least one atypical value out of individual limits (i.e. true positives/size of the rHuEpo group), and specificity was calculated given the portion of samples in the placebo group that presented no atypical value out of individual limits (i.e. true negatives/size of placebo group). For 41 of the 45 transcripts, within- and between-subject variances were estimated using the analysis of variance on the placebo data of log2 expression with subject as a random effect. A leave-one-out cross-validation procedure was used to minimise overfitting. The adaptive model was applied assuming universal components of variance and normality in the within-subject variations of the transcripts. A specificity level of 99% was chosen for all reference ranges returned by the adaptive model. Area under the Receiving Operating Characteristic (ROC) curve was computed on the percentiles at which HGB, OFF-score and all transcript sequences were falling in the distribution of sequences as returned by the adaptive model.

## Results

### Samples available for analysis

In the MDS, 343 out of 364 samples (i.e. 14 subjects × 2 trials × 13 time points), including 174 and 169 samples following rHuEpo and placebo injections, respectively, were collected and analysed using the QuantiGene Plex Assay (Affymetrix, Santa Clara, CA, USA). The average CV was 10.6% across all samples analysed (vs. 15% typical CV of the QuantiGene Plex Assay [32]). 171 (98.3% of 174) and 163 (96.4% of 169) samples under rHuEpo and placebo administration were available for the haematological analysis, respectively. 164 of 168 MDS exercise samples (i.e. 14 subjects × 2 trials × 6 exercise samples) were available for the QuantiGene Plex 2.0 Assay (Affymetrix, Santa Clara, CA, USA) analysis. The average CV was 16% across these samples. In the ATS elite runners, 66 of 72 samples (i.e. 12 subjects × 6 time points) obtained following altitude training and 22 of 27 samples (i.e. 9 subjects × 3 time points) obtained following sea-level training were processed using the QuantiGene Plex 2.0 Assay (Affymetrix, Santa Clara, CA, USA). 48 and 23 samples were available for HGB/HCT analysis in the elite runners and controls, and 37 and 21 samples for RET% calculation in the elite runners and controls, respectively. In the ATS elite rowers, 28 samples (i.e. 4 subjects × 7 time points) were available for the haematological and gene expression analyses. The average CVs were 8.4% and 9.8% across the samples from elite runners and rowers, respectively. Forty-one of the 45 transcripts exceeding the assay limit of detection were available for gene expression analysis in the MDS and ATS and 35 for MDS exercise-induced gene expression analysis, respectively.

### Haematological analysis

**Fig. 3:**
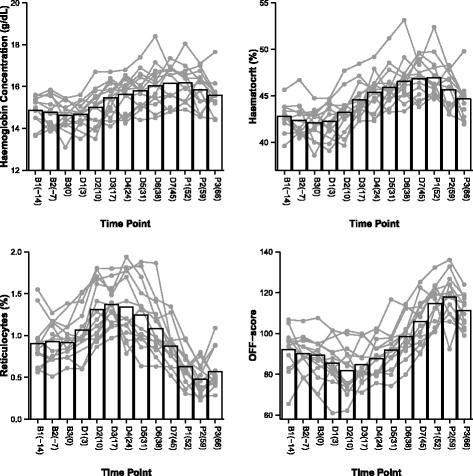
Haemoglobin concentration, haematocrit (%), reticulocytes (%) and OFF-score in subjects taken rHuEpo in the MDS. Data is displayed by means with corresponding individual changes over time. B: baseline; D: during rHuEpo administration; P: post rHuEpo administration. The number in the parentheses indicates the blood sampling day relative to the day of the first rHuEpo injection (i.e. B3)

In the MDS, both HGB concentration and HCT percentage were gradually increased throughout the rHuEpo administration relative to the baseline values and reached the maximum one week after the last injection (Holm-Bonferroni adjusted *p* < 0.05) (Fig. 3). RET% increased rapidly after the first injection and remained significantly elevated for 4 weeks (Holm-Bonferroni adjusted *p* < 0.05) (Fig. 3). The RET% was significantly lower compared to the baseline values (Holm-Bonferroni adjusted *p* < 0.05) throughout the post-rHuEpo phase (Fig. 3). The OFF-score showed an increasing trend during rHuEpo administration and significantly increased throughout the post phase (Holm-Bonferroni adjusted *p* < 0.05) (Fig. 3). No significant differences over time were found for the HGB and HCT parameters compared to baseline values in the placebo trial in the MDS, however, RET% were significantly increased at During 5 and Post 2 and while OFF-scores were significantly lower at During 4–7 and Post 1,2 in the placebo trial (Holm-Bonferroni adjusted *p* < 0.05). When comparing the blood data between the rHuEpo and placebo groups, similar trends and findings were obtained to those observed in the rHuEpo group. Haematological analysis in the ATS elite runners revealed a significant decrease in HGB during altitude (approximately 10 days at altitude), a significant increase in RET% and a significant decrease in OFF-scores post 2 weeks of sea-level training (Holm-Bonferroni adjusted *p* = 0.036, 0.02 and 0.002, respectively, see Additional files 2 and 3). No other significant differences were observed in these blood parameters over time at available time points (compared to the baseline), neither in the altitude group nor in the control group of the ATS elite runners (Holm-Bonferroni adjusted *p* > 0.05). Similarly, in the ATS elite rowers, no significant changes were observed over time in comparison to baseline values for the four haematological markers (see Additional file 4).

### Gene expression analysis

In the group of subjects following the rHuEpo injection in the MDS, differentially expressed genes were firstly selected based on the moderated F-statistic computed by the “eBayes” function implemented in the R limma package. Thirty-six out of the 41 genes exceeded the overall test of significance at the FDR adjusted *p* of 0.05. Of these 36 genes, 23 were selected when individual contrasts in gene expression revealed non-zero differences seven days after the last rHuEpo injection (i.e. Post 1) at FDR < 0.05. Subsequently, 17 of the 23 genes were found significantly altered in expression, exceeding a fold-change of 1.5 (FDR < 0.05), ten days (i.e. During 2) after the first injection. Among the 17 genes, 11 were consistently over-expressed from During 2 (Day 10) to During 5 (Day 31) and were then under-expressed throughout the post-administration stage, i.e. Post 1 (Day 52) to Post 3 (Day 66) (FDR < 0.05 with 1.5 fold-change threshold) (Table 1). Nine of the 11 genes, *ALAS2*, *BCL2L1*, *DCAF12*, *EPB42*, *GMPR*, *SELENBP1*, *SLC4A1*, *TMOD1* and *TRIM58* (Table 1 and Additional file 5), were common with the 34 transcripts previously identified and validated [23]. None of the 41 genes were differentially expressed in the placebo group over time (FDR > 0.15). When comparing the levels of gene expression in the rHuEpo group with that in the placebo group, 24 of the 41 genes were down-regulated at Post 1–3 (FDR < 0.05 with 1.5 fold-change threshold) (Table 2), overlapping the 9 genes aforementioned and no genes were differentially expressed in the “During” stage between the rHuEpo group and the placebo group (FDR > 0.17).

**Table 1:** 11 genes differentially expressed at During 2 to 5 and throughout the post rHuEpo administration in the MDS

Gene	During 2	During 3	During 4	During 5	Post 1	Post 2	Post 3	During 2	During 3	During 4	During 5	Post 1	Post 2	Post 3
Log2 FC								FC						
ALAS2	0.86	0.95	0.80	0.70	−0.82	−1.32	−1.18	1.81	1.93	1.75	1.63	−1.77	−2.50	−2.26
BCL2L1	0.71	0.81	0.79	0.69	−0.67	−0.88	−0.89	1.63	1.75	1.73	1.61	−1.59	−1.84	−1.85
DCAF12	0.60	0.78	0.73	0.66	−0.59	−0.90	−0.89	1.52	1.72	1.66	1.58	−1.50	−1.87	−1.86
EPB42	0.86	0.98	0.88	0.76	−0.89	−1.25	−1.08	1.82	1.97	1.84	1.69	−1.85	−2.37	−2.11
GMPR	0.81	0.94	0.79	0.68	−0.72	−0.94	−0.84	1.75	1.92	1.73	1.60	−1.65	−1.92	−1.79
OSBP2	0.96	1.08	1.05	1.00	−0.89	−1.16	−1.03	1.95	2.12	2.07	2.00	−1.85	−2.23	−2.04
SELENBP1	0.98	1.09	0.97	0.83	−0.90	−1.19	−1.05	1.97	2.13	1.96	1.77	−1.87	−2.29	−2.07
SLC4A1	0.89	1.08	0.96	0.82	−0.87	−1.18	−1.10	1.86	2.12	1.94	1.77	−1.83	−2.26	−2.14
TMOD1	0.65	0.89	0.76	0.61	−0.71	−1.06	−1.00	1.57	1.85	1.70	1.53	−1.64	−2.08	−2.01
TNS1	0.84	1.10	0.98	0.87	−0.94	−1.22	−1.10	1.79	2.14	1.97	1.83	−1.91	−2.34	−2.14
TRIM58	0.73	0.82	0.72	0.67	−0.74	−0.94	−0.97	1.66	1.76	1.65	1.59	−1.67	−1.92	−1.96
Uncorrected *P* val.								FDR						
ALAS2	0.00181	0.00055	0.00337	0.01011	0.00274	0.000003	0.00002	0.00778	0.00173	0.00767	0.02030	0.01443	0.00006	0.00008
BCL2L1	0.00180	0.00038	0.00048	0.00230	0.00317	0.00013	0.00009	0.00778	0.00171	0.00367	0.01678	0.01443	0.00031	0.00025
DCAF12	0.00912	0.00072	0.00157	0.00434	0.01090	0.00014	0.00012	0.01558	0.00193	0.00430	0.01678	0.02482	0.00031	0.00031
EPB42	0.00488	0.00139	0.00416	0.01333	0.00378	0.00007	0.00045	0.01052	0.00300	0.00877	0.02277	0.01551	0.00022	0.00092
GMPR	0.00165	0.00026	0.00200	0.00793	0.00479	0.00034	0.00110	0.00778	0.00152	0.00492	0.01835	0.01679	0.00064	0.00196
OSBP2	0.00024	0.00004	0.00006	0.00014	0.00071	0.00002	0.00009	0.00778	0.00079	0.00252	0.00591	0.01443	0.00008	0.00025
SELENBP1	0.00118	0.00030	0.00129	0.00603	0.00283	0.00011	0.00053	0.00778	0.00152	0.00407	0.01835	0.01443	0.00030	0.00103
SLC4A1	0.00848	0.00148	0.00478	0.01499	0.01016	0.00069	0.00126	0.01511	0.00303	0.00933	0.02458	0.02451	0.00124	0.00215
TMOD1	0.00520	0.00015	0.00105	0.00849	0.00218	0.000010	0.00002	0.01052	0.00152	0.00388	0.01835	0.01443	0.00008	0.00008
TNS1	0.00531	0.00026	0.00114	0.00376	0.00181	0.00007	0.00027	0.01052	0.00152	0.00388	0.01678	0.01443	0.00022	0.00058
TRIM58	0.00190	0.00051	0.00204	0.00450	0.00172	0.00009	0.00004	0.00778	0.00173	0.00492	0.01678	0.01443	0.00026	0.00014

*Log2 FC* log2 transformed fold-change, *FC* fold-change, *FDR* false discovery rate adjusted significance

**Table 2:** 24 genes responded differently (significantly down-regulated over Post 1, 2, 3) in the rHuEpo group relative to the placebo group in the MDS

	Log2 FC			FC			Uncorrected *P* val.			FDR		
Gene	Post 1	Post 2	Post 3	Post 1	Post 2	Post 3	Post 1	Post 2	Post 3	Post 1	Post 2	Post 3
ADIPOR1	−0.69	−1.17	−1.02	−1.61	−2.24	−2.03	0.00669	0.00001	0.00007	0.01958	0.00009	0.00048
ALAS2	−1.10	−1.78	−1.55	−2.14	−3.43	−2.93	0.00504	0.00001	0.00008	0.01720	0.00009	0.00048
BCL2L1	−0.97	−1.29	−1.12	−1.96	−2.44	−2.18	0.00269	0.00010	0.00050	0.01720	0.00026	0.00172
BPGM	−0.72	−1.11	−1.04	−1.64	−2.16	−2.05	0.00417	0.00002	0.00004	0.01720	0.00010	0.00048
CA1	−0.82	−1.28	−1.12	−1.76	−2.42	−2.18	0.01519	0.00022	0.00090	0.02967	0.00044	0.00223
CSDA	−0.80	−1.25	−1.12	−1.75	−2.39	−2.18	0.00791	0.00005	0.00022	0.01959	0.00020	0.00088
DCAF12	−0.77	−1.24	−1.05	−1.70	−2.36	−2.07	0.01941	0.00024	0.00144	0.03351	0.00045	0.00282
EPB42	−1.30	−1.76	−1.44	−2.47	−3.39	−2.72	0.00286	0.00009	0.00097	0.01720	0.00025	0.00223
FAM46C	−0.66	−1.12	−0.98	−1.58	−2.17	−1.98	0.00462	0.00000	0.00003	0.01720	0.00009	0.00048
FBXO7	−0.64	−1.02	−0.94	−1.56	−2.03	−1.91	0.00792	0.00003	0.00011	0.01959	0.00014	0.00049
FECH	−0.78	−1.20	−1.13	−1.72	−2.30	−2.18	0.00545	0.00003	0.00007	0.01720	0.00014	0.00048
GMPR	−1.06	−1.39	−1.11	−2.08	−2.62	−2.16	0.00391	0.00022	0.00249	0.01720	0.00044	0.00465
GUK1	−0.77	−1.26	−1.05	−1.71	−2.40	−2.07	0.01462	0.00010	0.00098	0.02967	0.00026	0.00223
KRT1	−1.16	−1.53	−1.22	−2.24	−2.89	−2.33	0.00181	0.00006	0.00110	0.01720	0.00021	0.00236
OSBP2	−0.78	−1.13	−0.99	−1.72	−2.19	−1.99	0.00820	0.00020	0.00086	0.01959	0.00043	0.00223
SELENBP1	−1.28	−1.68	−1.39	−2.43	−3.21	−2.62	0.00295	0.00014	0.00124	0.01720	0.00033	0.00254
SLC4A1	−1.27	−1.60	−1.37	−2.42	−3.03	−2.58	0.00860	0.00123	0.00476	0.01959	0.00209	0.00813
SNCA	−0.84	−1.47	−1.28	−1.79	−2.77	−2.42	0.00952	0.00001	0.00009	0.02054	0.00009	0.00048
STRADB	−0.85	−1.36	−1.20	−1.80	−2.56	−2.30	0.00438	0.00001	0.00006	0.01720	0.00009	0.00048
TMOD1	−0.99	−1.43	−1.22	−1.98	−2.69	−2.33	0.00303	0.00003	0.00025	0.01720	0.00014	0.00094
TNS1	−1.26	−1.63	−1.26	−2.40	−3.10	−2.39	0.00318	0.00020	0.00330	0.01720	0.00043	0.00589
TRIM58	−0.93	−1.25	−1.15	−1.91	−2.38	−2.22	0.00536	0.00027	0.00061	0.01720	0.00049	0.00181
UBXN6	−0.71	−1.23	−1.05	−1.64	−2.35	−2.07	0.01962	0.00009	0.00062	0.03351	0.00025	0.00181
YOD1	−0.59	−1.10	−1.04	−1.50	−2.14	−2.05	0.01831	0.00002	0.00004	0.03351	0.00011	0.00048

*Log2 FC* log2 transformed fold-change, *FC* fold-change, *FDR* false discovery rate adjusted significance

Seven genes (1 up-, *CD247* and 6 down-regulated, *BPGM*, *FECH*, *SNCA*, *STRADB*, *TRIM58* and *YOD1*) of the 35 were significantly altered immediately following 10 sprints of 10 s compared to baseline (i.e. pre exercise) before the rHuEpo injection and 2 genes (1 up-, *CD247* and 1 down-regulated, *LOC100130562*) after the last rHuEpo injection (FDR < 0.05 with 1.5 fold-change threshold) (see Additional file 6). One (up-regulated, *CD247*) and 16 (1 up-, *CD247* and 15 down-regulated, *ADIPOR1*, *BCL2L1*, *BPGM*, *CA1*, *DCAF12*, *FAM46C*, *FBXO7*, *FECH*, *OSBP2*, *SNCA*, *STRADB*, *TRIM58*, *UBXN6*, *YBX3* and *YOD1*) genes of the 35 were found significantly altered immediately after the repeated sprint tests in comparison to pre-exercise gene expression before and after the placebo injection, respectively (FDR < 0.05 with 1.5 fold-change threshold) (see Additional file 6). No significant changes were found post 30 min of exercise vs. pre exercise in either rHuEpo or placebo trials. When comparing exercise gene expression changes before-after rHuEpo or placebo injections, no differences were observed, neither immediately nor 30 min post exercise (FDR > 0.93). Furthermore, there were no significant changes in transcription following exercise when comparing the rHuEpo group vs. the placebo group (FDR > 0.70).

In the 12 elite runners, 28 out of the 41 genes exceeded an overall F-test significance at the FDR adjusted *p* of 0.05 following altitude training (see Additional file 7, green section). The remaining 13 genes demonstrated non-significant changes in gene expression (F-test FDR > 0.05; see Additional file 7). Following pairwise comparisons, trends towards 5% FDR were observed for 20 genes (out of the 28) down-regulated one week after returning from altitude and 13 genes (out of the 20) were up-regulated approximately 10 days after reaching altitude (FDR > 0.059 and 0.064, respectively; see Additional file 7). Of the 13 genes, *ALAS2*, *BCL2L1*, *DCAF12*, *SLC4A1 TMOD1* and *TRIM58* (FDR > 0.059 with the fold-change varied from 1.39 to 1.63, see Additional files 7 and 8) were in common with the 9 MDS genes. In the elite runners, no genes were differentially expressed in the control group or responded differently over time in the altitude group relative to the control group post 2 and 4 weeks of altitude or sea-level training (FDR > 0.20 and 0.60, respectively). In the 4 elite rowers, no genes responded differently following 2-week altitude in conjunction with adaptation to simulated altitude when compared with the averaged baseline.

### Adaptive model analysis

In the MDS, using 13 samples per subject collected at weekly intervals including 3 weekly samples post-supplementation, rHuEpo use was identified in 13 out of 14 subjects and without any false positives, for a specificity of 99% in the ABP using the haematological markers of HGB and OFF-score (Table 3); 41 out of the 45 were amenable to analysis using the adaptive model. A higher between- compared to within-subject variability was observed across the examined genes (averaged variance: 0.21 vs. 0.13; Table 3). Fifteen transcripts showed a sensitivity of ≥93% and a specificity of ≥71%, with the ROC area ≥ 0.92 (Table 3). *BCL2L1* and *CSDA* were the genes with the highest sensitivity (93%) and specificity (93%), with the ROC area of 0.96 and 0.98, respectively (Table 3). In the MDS differential gene expression analysis, the *BCL2L1* gene was significantly expressed at During 2–5 and Post 1–3 in the rHuEpo group (Table 1, FDR < 0.05 and fold-change >1.5) and at Post 1–3 in the rHuEpo group vs. the placebo group (Table 2, FDR < 0.05 and fold-change >1.5); the *CSDA* gene was significantly down-regulated at Post 1–3 (FDR < 0.05 and fold-change >1.5) in the rHuEpo group vs. the placebo group (Table 2). HGB concentration (g·L^−1^), OFF-score and gene expression changes (of the 41 transcripts) across 28 subjects (14 subjects × 2 trials) obtained from the adaptive model and ROC curves are provided in Additional files 9, 10 and 11.

**Table 3:** The adaptive model analysis summarising the within- and between-subject variances, sensitivity, specificity and ROC area for the HGB concentration, OFF-score and 41 transcripts analysed in the MDS

	Mean	Within-subject variance	Between-subject variance	Sensitivity (%)	Specificity (%)	ROC area
HGB (g·L ^-1^)	150	29	36	79	93	0.87
OFF-score	92	51	62	93	93	0.93
ADIPOR1	3.33	0.098	0.085	86	79	0.97
ALAS2	3.09	0.204	0.282	100	79	0.97
BCL2L1	1.33	0.159	0.202	93	93	0.96
BPGM	2.74	0.107	0.087	93	71	0.98
CA1	1.28	0.159	0.190	93	79	0.97
CCR7	−0.15	0.092	0.155	29	93	0.78
CD247	−0.66	0.059	0.046	43	71	0.62
CD3D	0.30	0.061	0.048	43	64	0.63
CSDA	2.68	0.136	0.150	93	93	0.98
DCAF12	2.18	0.146	0.198	93	79	0.98
EEF1D	1.75	0.026	0.027	43	64	0.63
EPB42	−0.91	0.243	0.448	93	86	0.96
FAM46C	3.74	0.083	0.061	93	79	0.95
FBXO7	3.54	0.079	0.075	79	79	0.92
FECH	1.34	0.142	0.119	86	71	0.95
GMPR	0.23	0.181	0.281	86	86	0.93
GUK1	0.51	0.130	0.228	86	79	0.95
GYPE	−4.87	0.145	0.282	79	93	0.92
HBD	0.20	0.243	0.885	64	64	0.95
KRT1	−1.31	0.327	1.229	86	71	0.86
LEF1	0.37	0.083	0.129	36	93	0.68
LOC100130562	2.78	0.032	0.027	50	71	0.63
LOC286444	0.73	0.034	0.049	50	79	0.69
MIF	−0.15	0.043	0.021	36	71	0.62
OSBP2	0.61	0.205	0.206	93	79	0.97
PITHD1	0.20	0.125	0.183	86	86	0.92
RBM38	0.44	0.150	0.566	79	79	0.89
RNF213	0.76	0.102	0.062	36	79	0.42
SELENBP1	0.63	0.240	0.401	93	79	0.92
SGK223	−2.81	0.064	0.042	36	79	0.60
SKAP1	−1.52	0.055	0.052	43	79	0.61
SLC4A1	1.36	0.213	0.595	93	86	0.96
SNCA	0.87	0.172	0.154	86	71	0.96
STRADB	2.29	0.136	0.145	93	86	0.99
TMOD1	−0.57	0.188	0.163	93	71	0.95
TNS1	−2.05	0.234	0.306	93	86	0.96
TPRA1	−2.43	0.011	0.010	50	79	0.67
TRIM58	2.23	0.156	0.199	86	71	0.98
UBXN6	2.06	0.107	0.209	93	71	0.98
VEGFB	−1.62	0.045	0.039	57	64	0.67
YOD1	1.48	0.137	0.050	71	71	0.90

*ROC* receiving operating characteristic

## Discussion

Twenty-four of the 41 genes showed a significant and long lasting down-regulation following the last rHuEpo injection in the MDS given a fold-change of 1.5 and 5% FDR spanning the post rHuEpo stage for 3 weeks (Table 2). This prolonged detection window in terms of the long lasting effect and the stability of the gene markers collected using the Tempus™ Blood RNA method is promising and will undoubtedly improve the efficiency of rHuEpo detection given the substantially shorter detection duration of 36 h for improved analytical quality when using the current haematological markers [8]. Fifteen of the 24 genes showed a high sensitivity (≥ 93%) with specificity equal to or above 71% (Table 3). Particularly, the *BCL2L1* and *CSDA* exhibited the highest sensitivity (93%) and specificity (93%) amongst the 15 genes (Table 3). The majority of the 9 genes that were consistently expressed during and post rHuEpo in the MDS rHuEpo group, namely *ALAS2*, *BCL2L1*, *DCAF12*, *EPB42*, *GMPR*, *SELENBP1*, *SLC4A1*, *TMOD1* and *TRIM58* were associated with erythrocyte membrane structure and red blood cell metabolism; these 9 genes were in common with the 34 transcripts previously identified and validated [23], while 7 of the 9 genes were in common with the 15 genes showing high sensitivity and specificity. The major spliced mRNA isoform of the *ALAS2* has significant impact on erythroid heme biogenesis and hemogobin formation [33]. Erythrocyte survival is suppressed by the BH3 peptide through antagonizing Bcl-X(L) [34]. EPB42 deficiency causes hereditary spherocytosis, leading to chronic haemolytic anemia with abnormally shaped erythrocytes [35]. The *EPB42* may be involved in the regulation of erythrocyte shape and mechanical properties [36]. The *SELENBP1* is thought to play a role in rapid cell outgrowth by determining the direction of the outgrowth and the synthesis of actin filaments [37]. There are two structurally and functionally distinct versions of the protein encoded by the *SLC4A1* gene – the N-terminal 40 kDa cytoplasmic domain attaches to the red cell skeleton by binding ankyrin to maintain the structure of red blood cells and the C-terminal 50 kDa membrane domain is responsible for the transport of anions, by facilitating the exchange of chloride and bicarbonate across the plasma membrane of erythrocytes [38–40]. The TMOD1 protein also influences the structure of the erythrocyte membrane skeleton by regulating tropomyosin [41]. The *GMPR* gene is mapped to chromosome 6p23 [42] and maintains the intracellular balance of guanine and adenine nucleotides [43]. It was previously reported that the human red cell glucose-6-phosphate dehydrogenase is encoded by the chromosome 6- and chromosome X-encoded genes [44] but subsequently disproved by other studies [45, 46]. The *DCAF12* and *TRIM58* as well as *CSDA* genes are associated with terminal erythroid differentiation, red blood cell count and red blood cell interactome networks, respectively [47–49]. As stated previously, 5 genes (i.e. *PFN1*, *C13orf15*, *TSTA3*, *RPL41* and *TOMM40*) were identified by Varlet-Marie et al. using SAGE following administration of the erythropoiesis-stimulating agent (ESA) darbepoetin alpha and high doses and microdoses of rHuEpo [19]. However, none of these genes overlapped with the molecular signature of rHuEpo doping identified and validated in our previous and current studies. Differences in study design including the specific drugs, drug administration methods, sample size and detection platforms, are all likely factors contributing to these results, emphasising further the need for rigorous replication of any markers identified in a single study.

The identification of a similar panel of genes associated with the structure and function of red blood cells identified in the present study and those of other clinical groups (see ref. [33–49]) is encouraging and should assist the development of targeted therapy to treat patients with blood disorders. There are widespread applications utilising rHuEpo in clinical settings such as the treatment of patients with anaemia of chronic renal disease, improving quality of life in cancer patients and minimising the transfusion requirement [50], but not all patients respond effectively to treatment with rHuEpo. The transcriptomic markers identified in healthy individuals administered with rHuEpo in our previous and current studies represent useful targets to investigate the signal transduction pathways activated by erythropoietin and its receptor for improved therapeutic use of ESA and rHuEpo. With reference to anti-doping, the whole-blood trancriptomic markers discovered reflect closely the RET% changes over time (Fig. 3). This is consistent with the finding that a large amount of mRNA species in whole blood originate from reticulocytes, evidenced by the separate clustering of whole blood samples treated with RNase H and leukocyte samples in microarray analysis [51]. New approaches that are able to interrogate the whole transcriptome with improved dynamic range for adequate quantification (e.g. RNA-seq) and preferably in leucocytes populations in a sufficient number of participants are warranted in future anti-doping transcriptomic studies and especially those involving the manipulation of red blood cells to improve further the detection of blood doping using molecular markers. Approaches enabling the interrogations of whole genome, transcriptome and metabolome have the increased capacity to measure rapidly and in the near future, inexpensively a large number of molecular signatures, which will collectively aid the decision making during identifying and differentiating numerous doping substances and methods by ABP experts when reviewing passports. For example, one of the transcripts validated in the present study is the *BPGM* gene (Table 2), encoding the 2–3 BPG. It is well known that blood doping can affect 2–3 BPG metabolism and is therefore closely monitored by the WADA ABP Expert Panel. In addition, the only subject evading detection by the haematological model of ABP in the present study (see Additional files 9 and 10, subject R) had a low mean cell volume and mean cell haemoglobin, indicating iron deficiency or a defect in iron metabolism and particularly that the subject participated in the present study is non-responsive to iron supplementation. However, 25 of the 41 genes included in the adaptive model analysis identified rHuEpo use in the same subject (see Additional file 11, subject 9). This illustrates the need for a holistic approach to drug detection; one based on the inclusion of a variety of parameters that provide information on related pathways and metabolism (e.g. iron, transferring and total iron binding capacity). Notably, the large-scale omics studies combined with the ABP are anticipated to be able to dramatically improve doping detection. It is also important to note that the potential for the validated transcripts in the present study to identify the rHuEpo use is high both when the transcripts are used alone (Tables 1 and 2; 24 genes exceeding a fold-change of 1.5 at 5% FDR post rHuEpo injections) or in combination with the current ABP (Table 3; 20 genes with a ROC area > 0.95), emphasising the need to understand erythropoiesis using a systems biology approach.

The *CD247* gene was significantly up-regulated exceeding a fold-change of 1.5 at 5% FDR immediately following exercise when compared to the baseline before and after rHuEpo/placebo, respectively; but this response rapidly subsided 30 min after exercise. The *CD247* encodes the T-cell receptor zeta, which is a component of the T-cell receptor-CD3 complex [52]. Low expression of the gene may relate to impaired immune response [53, 54]. This current observation is in line with previous studies indicating acute inflammatory response following acute resistance exercise [55, 56] and the subsequent anti-inflammatory response is to prevent the development of chronic inflammation [21, 57]. Six and fifteen genes were significantly down-regulated (6 is included in the 15) immediately following exercise before rHuEpo and after placebo, respectively (FDR < 0.05 and fold-change >1.5). Among these genes, only *TRIM58* overlaps the 9 MDS genes. *TRIM58* specifically expresses during late erythroid maturation, coinciding with enucleation and dynein loss [58]. The suppression of *TRIM58* relates to reduced enucleation [58]. *TRIM58* gene polymorphisms associate with the circulating erythrocyte size and number [48, 59]. Previous studies reported an increased number of leukocytes immediately following heavy exercise [21, 22], whereas the reduced level of *TRIM58* following the repeated sprint test in the present study may reflect reduced proportions of reticulocytes in accordance with previous research [58]. This modified Wingate test did not reveal significant gene expression changes using the 35 transcripts when comparing before with after rHuEpo/placebo, or rHuEpo vs. placebo. These results, although preliminary, argue against exercise/training being a confounder as all gene expression alterations post strenuous exercise were restored after 30 min and well within the two hours stipulated by WADA before a blood sample can be obtained for anti-doping purposes [60]. Nevertheless, the explicit role of exercise needs to be further investigated not only in whole blood but also peripheral blood cells in order to better understand the molecular adaptations to both acute and chronic exercise (i.e. exercise training) and avoid confounding the analysis.

Previous studies have shown that short or prolonged residency at high altitude stimulates the secretion of Epo from the kidney [61–64]. For example, elevated levels of Epo are detected as early as 8 h after arrival at high altitude and reaches a peak 24 h after arrival [61]. These higher levels of Epo are also maintained throughout a period of high altitude exposure (e.g., ranging from 11 days to 4 weeks at approximately 3500 m or above [61, 64]). In the present ATS, 13 out of the 41 genes failed to show statistically significant changes at the transcriptional level following approximately 2-week training at moderate altitude in 12 elite runners given a F-test FDR > 0.05. Six of the remaining 28 genes that tended to be stimulated by altitude were in common with the 9 MDS genes (FDR > 0.059 with the fold-change varied from 1.39 to 1.63 during and after one week of altitude training; see Additional files 7 and 8). Among these 6 genes, *SLC4A1* showed a tendency towards a 1.63-fold down-regulation (FDR = ~ 0.059) one week after altitude training and *TMOD1* a tendency for a 1.50-fold up-regulation (FDR = ~ 0.064) after ten days of altitude exposure; none of the other genes exceeded the 1.5 fold-change cut-off (FDR > 0.059) (see Additional file 7). In comparison, in the MDS rHuEpo group, *DCAF12* showed a least 1.52-fold up-regulation (FDR = ~ 0.016) ten days after the first rHuEpo injection (i.e. During 2) and this level of expression was maintained for 4 weeks for all 6 genes (i.e. During 2–5, FDR < 0.05, Table 1). In addition, there was at least 1.50-fold down-regulation (FDR = ~ 0.025) in *DCAF12* one week after the last rHuEpo injection (i.e. Post 1) with this level of change lasting throughout the 3 weeks for all 6 genes (i.e. Post 1–3, FDR < 0.05, Table 1), with majority of the 6 genes approaching or exceeding a 2 fold-change at the post rHuEpo stage (Table 1). There was also a more pronounced gene expression response in these 6 genes post rHuEpo administration compared to the placebo group (FDR < 0.05, Table 2), with an approximate 2.5 fold-change in *ALAS2*, *SLC4A1* and *TMOD1* 2 and 3 weeks after the last injections (i.e. Post 2, 3, Table 2). As stated previously, the observed patterns of change in gene expression in MDS reflect closely the RET% changes over time (Fig. 3). In comparison, haematological analysis in the ATS elite runners revealed a small but significant decrease in HGB during altitude (approximately 10 days at altitude) (FDR = 0.036, see Additional file 2). No genes were differentially expressed in the control group or responded differently over time in the altitude group relative to the control group and this trend was in general agreement with the haematological results that showed no change over time despite exposure to altitude. The relatively long lasting effects and high magnitude of changes of the validated transcriptional markers of rHuEpo compared with the gene expression changes of the same markers (i.e. the 6 genes) in the ATS elite runners provide strong evidence in favour of applying such markers, alongside current anti-doping strategies to detect blood doping. The finding of no change in gene expression or in the measured haematological variables in the four elite rowers most likely reflects the limited exposure to moderate altitude, the small sample size and the typical inter-individual variation (see additional file 4). An altitude study with extended exposure (for 4 weeks at least) at high altitude (~ 3000 m) involving the analysis of both whole blood and peripheral blood for omics marker identification is required to better understand the molecular adaptations to altitude as compared to blood doping. Only then can the molecular response to altitude training be excluded, or if needed, integrated in the ABP along with other confounders to detect blood doping. Assessing and determining other confounding variables influencing the biological and analytical variability of the gene markers through altered erythropoiesis presents a critical next step to enhance the specificity and sensitivity of current ABP for unbiased detection of blood doping in conjunction with a systems biology approach.

## Conclusions

In conclusion, several human whole-blood transcriptional signatures signifying predominantly altered red blood cell production were identified following rHuEpo injections ranging from high doses to microdoses. These findings support the use of molecular markers as potential biomarkers with an improved detection window and high sensitivity and specificity for developing the transcriptionally-enhanced ABP model for detecting blood doping. Collectively, the findings of the present study, interpreted in the context of the latest omics research, are encouraging and suggest a systems biology approach combining various omics signatures from genomics, transcriptomics, proteomics and metabolomics will inevitably provide a deeper understanding of the effects of erythropoietic stimulating agents on erythropoiesis with unparalleled potential to improve current drug detection strategies with particular reference to blood doping. However, continuous and rigorous efforts will be required to determine, accommodate and possibly eliminate other confounding effects on blood doping.

## Additional files

Additional file 1: List of the 50 genes for the QuantiGene Plex Assay analysis in the MDS and ATS. (DOC 67 kb)

Additional file 2: Haemoglobin concentration (g·dL^−1^), haematocrit (%), reticulocytes (%) and OFF-score in response to altitude training in the 11 ATS elite runners. Data is displayed by means with corresponding individual changes over time. B(−7), D(10), P(16), P(30) and P(42) represent pre, during, 48-h-, 1-week-, and 4-week-post altitude exposure, respectively. (PDF 7 kb)

Additional file 3: Haemoglobin concentration (g·dL^−1^), haematocrit (%), reticulocytes (%) and OFF-score changes in the 7 ATS elite runners at the sea level. Data is displayed by means with corresponding individual changes over time. B(−7), P(30) and P(42) represent pre, 1-week-, and 4-week-post altitude exposure, respectively. (PDF 6 kb)

Additional file 4: Haemoglobin concentration (g·dL^−1^), haematocrit (%), reticulocytes (%) and OFF-score in the four ATS elite rowers. Data is displayed by means with corresponding individual changes over time. B1(−14) and B2(−10): 14 and 10 days prior to 5-day simulated altitude, respectively; D1(5), D2(22), P1(29), P2(33) and P3(39): 5, 22, 29, 33 and 39 days relative to the first day of the simulated altitude prior to the natural altitude exposure. (PDF 7 kb)

Additional file 5: Individual expression of the *ALAS2*, *BCL2L1*, *DCAF12*, *EPB42*, *GMPR*, *SELENBP1*, *SLC4A1*, *TMOD1* and *TRIM58* genes over time in response to rHuEpo in the MDS, respectively. (PDF 23 kb)

Additional file 6: Genes differentially expressed immediately following the repeated sprint tests vs. baseline prior to and after the rHuEpo/placebo injection in the MDS, respectively. (XLS 21 kb)

Additional file 7: Summary of gene expression changes over time in response to altitude exposure in the 12 ATS elite runners for the 41 transcripts. (XLS 37 kb)

Additional file 8: Individual expression of the *ALAS2*, *BCL2L1*, *DCAF12*, *SLC4A1*, *TMOD1* and *TRIM58* genes over time in response to altitude exposure in the 12 ATS elite runners, respectively. B(−7), D(10), P(16), P(21), P(30) and P(42) represent pre, during, 48-h-, 1-week-, 2-week- and 4-week-post altitude exposure, respectively. (PDF 9 kb)

Additional file 9: Adaptive model analysis and ROC curve in HGB concentration (g·L^−1^) across 28 subjects (14 subjects × 2 trials) in the MDS. Red lines: individual limits as determined by the adaptive model for a specificity of 99%; blue line: actual test results. Subject A, D, F. G, J, K, M, P, R, S, U, X, Z and AA participated in the rHuEpo trial; subject B, C, E, H, I, L, N, O, Q, T, V, W, Y and BB participated in the placebo trial. X-axis: 13 time points and y-axis: HGB concentrations. (ZIP 88 kb)

Additional file 10: Adaptive model analysis and ROC curve in OFF-score across 28 subjects (14 subjects × 2 trials) in the MDS. Red lines: individual limits as determined by the adaptive model for a specificity of 99%; blue line: actual test results. Subject A, D, F. G, J, K, M, P, R, S, U, X, Z and AA participated in the rHuEpo trial; subject B, C, E, H, I, L, N, O, Q, T, V, W, X and AB participated in the placebo trial. X-axis: 13 time points and y-axis: OFF-scores. (ZIP 87 kb)

Additional file 11: Adaptive model analysis and ROC curve in gene expression changes of the 41 transcripts across 28 subjects (14 subjects × 2 trials) in the MDS. Red lines: individual limits as determined by the adaptive model for a specificity of 99%; blue line: actual test results. The first 1–14 graphs: subjects participated in the rHuEpo trial and following 15–28 graphs: the same subjects participated in the placebo trial. X-axis: 13 time points and y-axis: log2 gene expression. (ZIP 1076 kb)
