# Supplementary material for: Validation of whole-blood transcriptome signature during microdose recombinant human erythropoietin (rHuEpo) administration
Source: BMC Genomics. 2017 Nov 14;18(Suppl 8):817. doi: 10.1186/s12864-017-4191-7 (PMC5688496; doi:10.1186/s12864-017-4191-7)
Supplement: Supplementary file 1 — List of the 50 genes for the QuantiGene Plex Assay analysis in the MDS and ATS. (DOC 67 kb) [file 12864_2017_4191_MOESM1_ESM.doc]

| **Additional file 1.** List of the 50 genes for the QuantiGene Plex Assay analysis in the MDS and ATS. | | | |
| --- | --- | --- | --- |
| **Gene Symbol** | **Gene Name** | **Type** |  |
| **ACTB** | Actin, beta | HK |  |
| **ACTR10** | Actin-related protein 10 homolog (S. cerevisiae) | HK |  |
| **ADIPOR1** | Adiponectin receptor 1 | Target |  |
| **ALAS2** | 5'-aminolevulinate synthase 2 | Target |  |
| **BCL2L1** | BCL2-like 1 | Target |  |
| **BPGM** | 2,3-bisphosphoglycerate mutase | Target |  |
| **CA1** | Carbonic anhydrase I | Target |  |
| **CCR7** | Chemokine (C-C motif) receptor 7 | Target |  |
| **CD247** | CD247 molecule | Target |  |
| **CD3D** | CD3d molecule, delta (CD3-TCR complex) | Target |  |
| **CSDA** | Cold shock domain protein A | Target |  |
| **DCAF12** | DDB1 and CUL4 associated factor 12 | Target |  |
| **EEF1D** | Eukaryotic translation elongation factor 1 delta (guanine nucleotide exchange protein) | Target |  |
| **EPB42** | Erythrocyte membrane protein band 4.2 | Target |  |
| **FAM46C** | Family with sequence similarity 46, member C | Target |  |
| **FBXO7** | F-box protein 7 | Target |  |
| **FECH** | Ferrochelatase | Target |  |
| **GMPR** | Guanosine monophosphate reductase | Target |  |
| **GUK1** | Guanylate kinase 1 | Target |  |
| **GYPE** | Glycophorin E (MNS blood group) | Target |  |
| **HBD** | Hemoglobin, delta | Target |  |
| **HBE1** | Hemoglobin, epsilon 1 | Target |  |
| **KRT1** | Keratin 1 | Target |  |
| **LEF1** | Lymphoid enhancer-binding factor 1 | Target |  |
| **LOC100130562** | LOC100130562 | Target |  |
| **LOC286444** | LOC286444 | Target |  |
| **MIF** | Macrophage migration inhibitory factor (glycosylation-inhibiting factor) | Target |  |
| **MRFAP1** | Mof4 family associated protein 1 | HK |  |
| **OSBP2** | Oxysterol binding protein 2 | Target |  |
| **PITHD1** | PITH (C-terminal proteasome-interacting domain of thioredoxin-like) domain containing 1 | Target |  |
| **PPIB** | Peptidylprolyl isomerase B (cyclophilin B) | HK |  |
| **RAB11A** | RAB11A, member RAS oncogene family | HK |  |
| **RBM38** | RNA binding motif protein 38 | Target |  |
| **RNF213** | Ring finger protein 213 | Target |  |
| **ROPN1B** | Ropporin, rhophilin associated protein 1B | Target |  |
| **SELENBP1** | Selenium binding protein 1 | Target |  |
| *continued on next page*  *Continued on next page* | | | |
| **Gene Symbol** | **Gene Name** | **Type** |  |
| **SERPINA13** | Serpin peptidase inhibitor, clade A (alpha-1 antiproteinase, antitrypsin), member 13 (pseudogene) | Target |  |
| **SGK223** | Homolog of rat pragma of Rnd2 | Target |  |
| **SKAP1** | Src kinase associated phosphoprotein 1 | Target |  |
| **SLC4A1** | Solute carrier family 4, anion exchanger, member 1 (erythrocyte membrane protein band 3, Diego blood group) | Target |  |
| **SLC6A10P** | Solute carrier family 6 (neurotransmitter transporter, creatine), member 10 (pseudogene) | Target |  |
| **SNCA** | Synuclein, alpha (non A4 component of amyloid precursor) | Target |  |
| **STRADB** | STE20-related kinase adaptor beta | Target |  |
| **TMOD1** | Tropomodulin 1 | Target |  |
| **TNS1** | Tensin 1 | Target |  |
| **TPRA1** | Transmembrane protein, adipocyte asscociated 1 | Target |  |
| **TRIM58** | Tripartite motif-containing 58 | Target |  |
| **UBXN6** | UBX domain protein 6 | Target |  |
| **VEGFB** | Vascular endothelial growth factor B | Target |  |
| **YOD1** | YOD1 deubiquitinase | Target |  |
| HK: housekeeping gene | | | |
