# Supplementary figures and images for: Validation of whole-blood transcriptome signature during microdose recombinant human erythropoietin (rHuEpo) administration
Source: BMC Genomics. 2017 Nov 14;18(Suppl 8):817. doi: 10.1186/s12864-017-4191-7 (PMC5688496; doi:10.1186/s12864-017-4191-7)

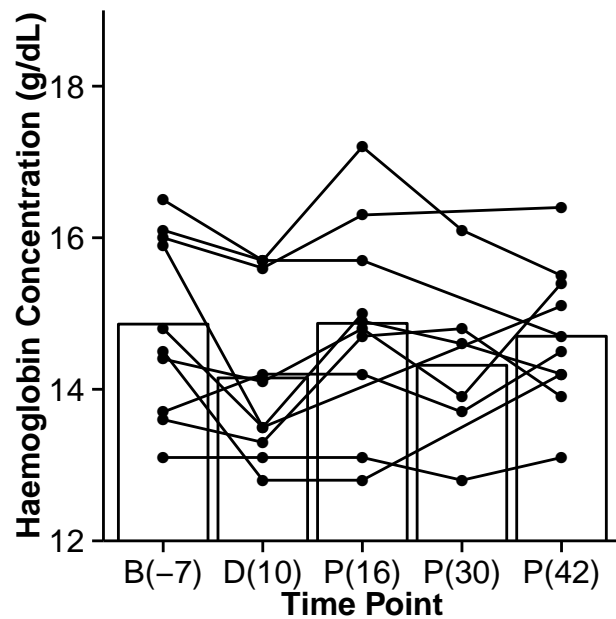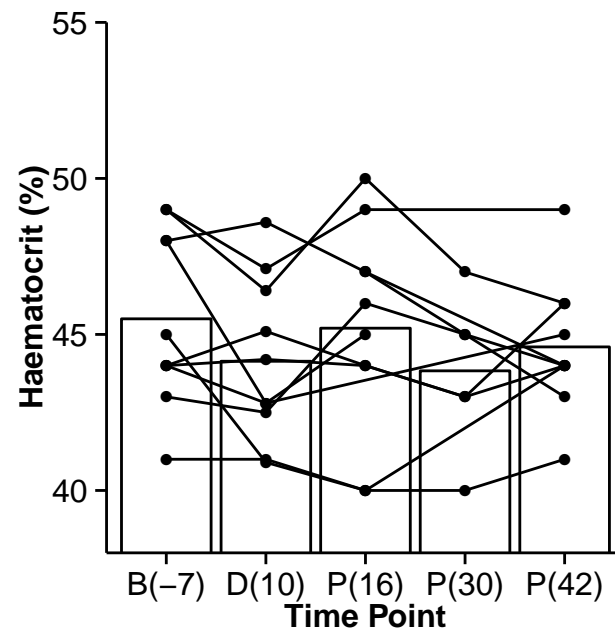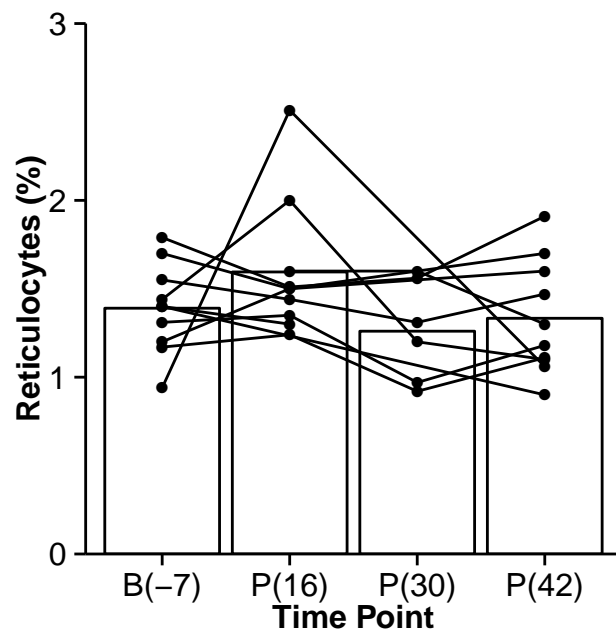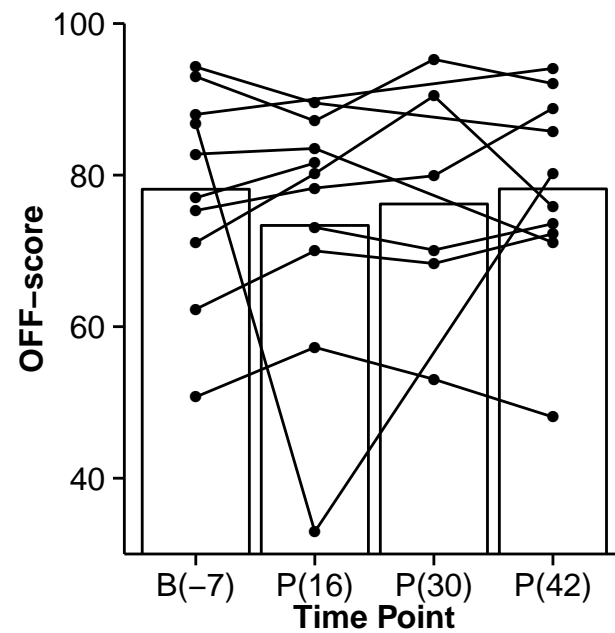

Supplement: Supplementary file 2 — Haemoglobin concentration (g·dL−1), haematocrit (%), reticulocytes (%) and OFF-score in response to altitude training in the 11 ATS elite runners. Data is displayed by means with corresponding individual changes over time. B(−7), D(10), P(16), P(30) and P(42) represent pre, during, 48-h-, 1-week-, and 4-week-post altitude exposure, respectively. (PDF 7 kb) [file 12864_2017_4191_MOESM2_ESM.pdf]

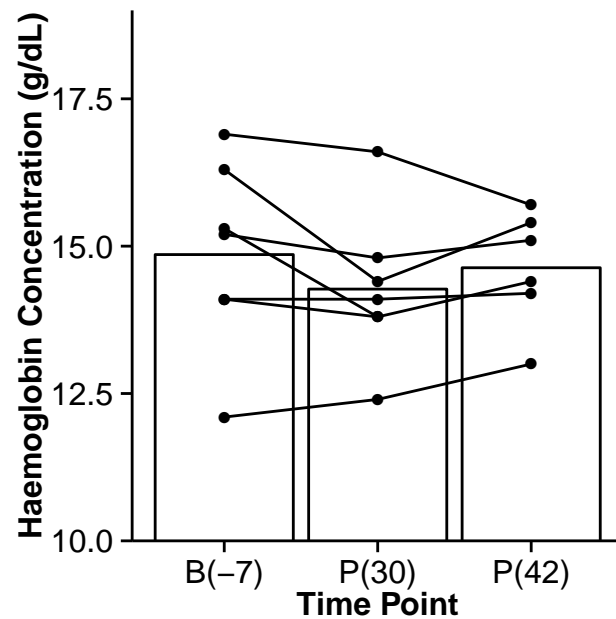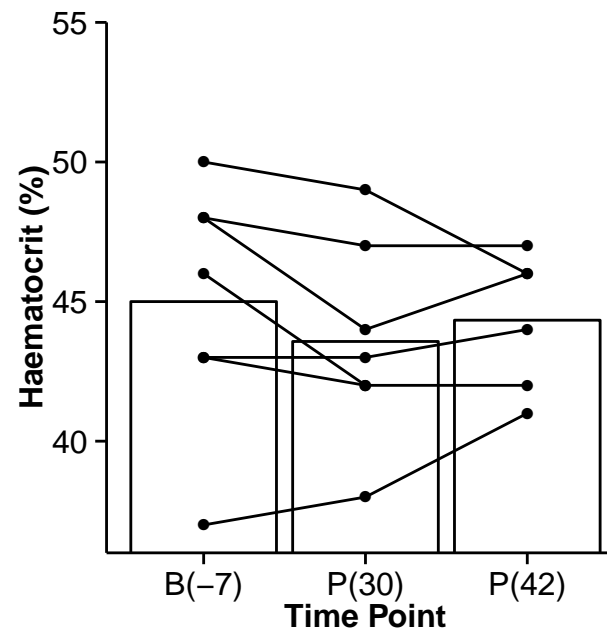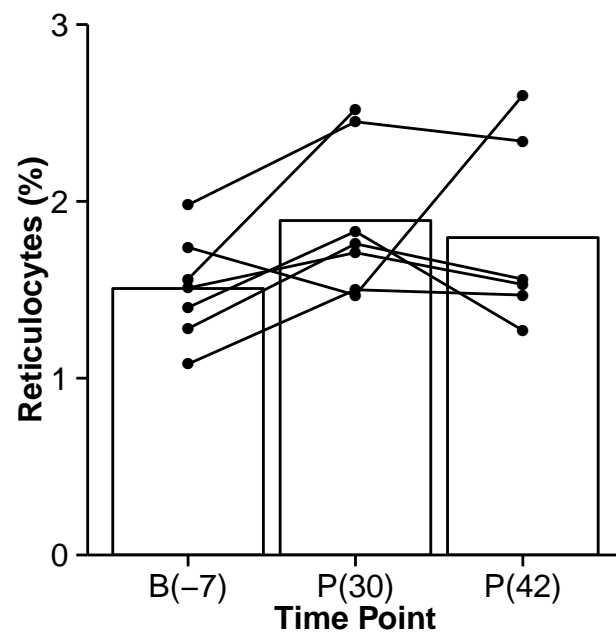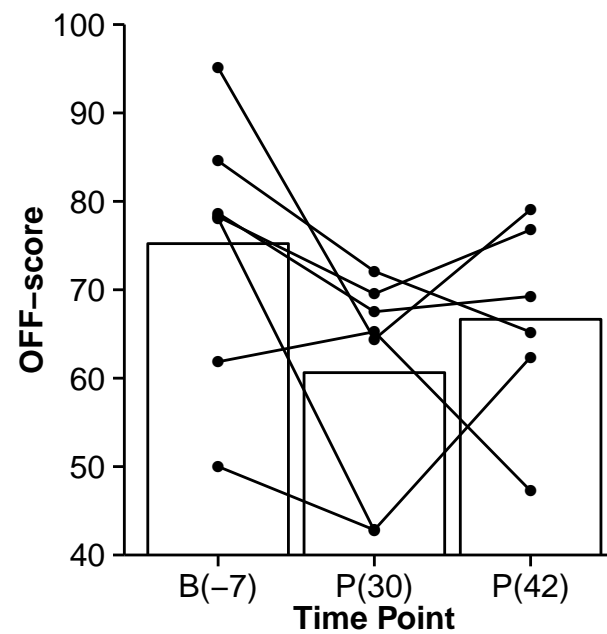

Supplement: Supplementary file 3 — Haemoglobin concentration (g·dL−1), haematocrit (%), reticulocytes (%) and OFF-score changes in the 7 ATS elite runners at the sea level. Data is displayed by means with corresponding individual changes over time. B(−7), P(30) and P(42) represent pre, 1-week-, and 4-week-post altitude exposure, respectively. (PDF 6 kb) [file 12864_2017_4191_MOESM3_ESM.pdf]

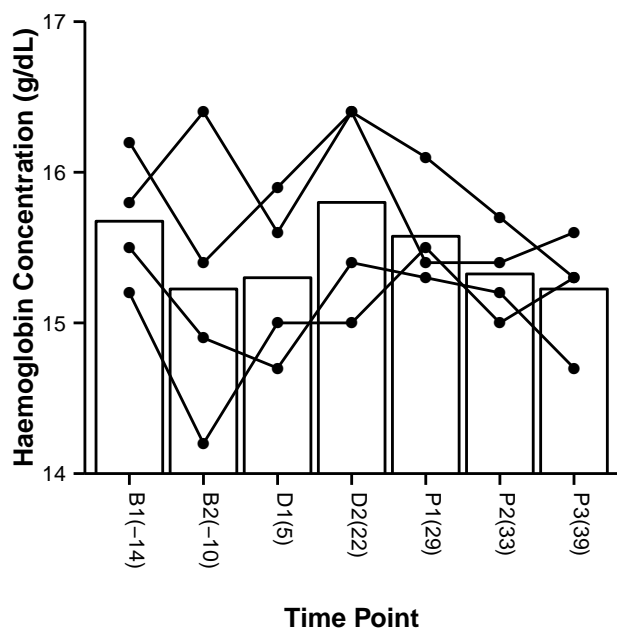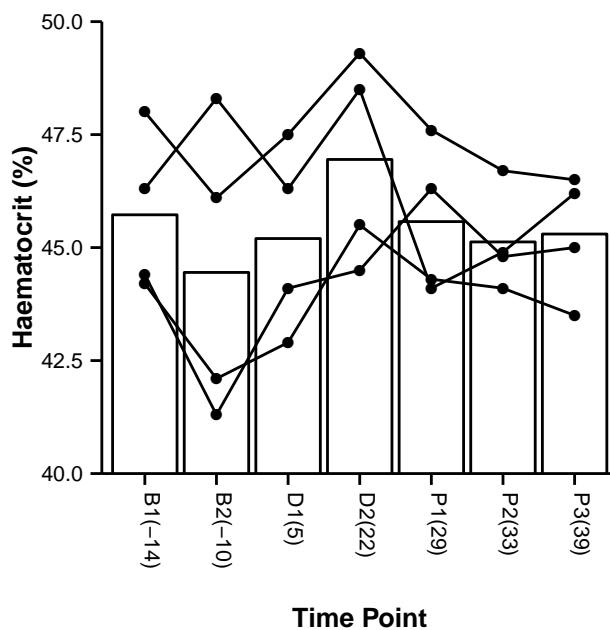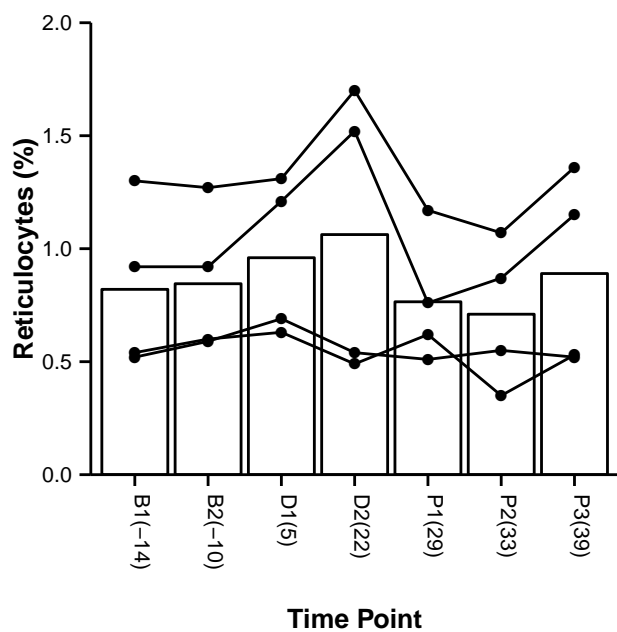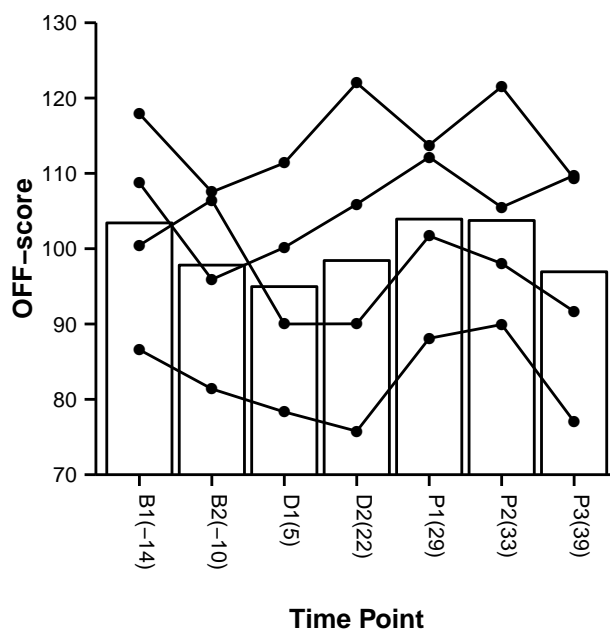

Supplement: Supplementary file 4 — Haemoglobin concentration (g·dL−1), haematocrit (%), reticulocytes (%) and OFF-score in the four ATS elite rowers. Data is displayed by means with corresponding individual changes over time. B1(−14) and B2(−10): 14 and 10 days prior to 5-day simulated altitude, respectively; D1(5), D2(22), P1(29), P2(33) and P3(39): 5, 22, 29, 33 and 39 days relative to the first day of the simulated altitude prior to the natural altitude exposure. (PDF 7 kb) [file 12864_2017_4191_MOESM4_ESM.pdf]

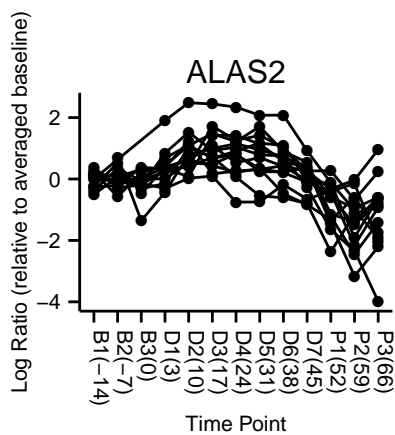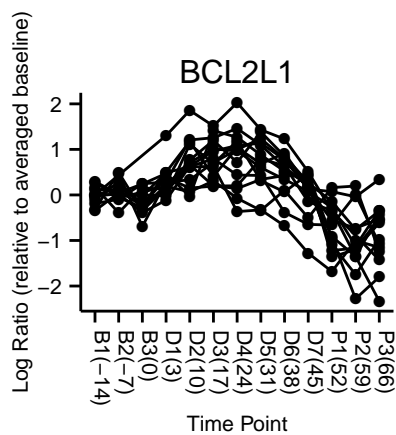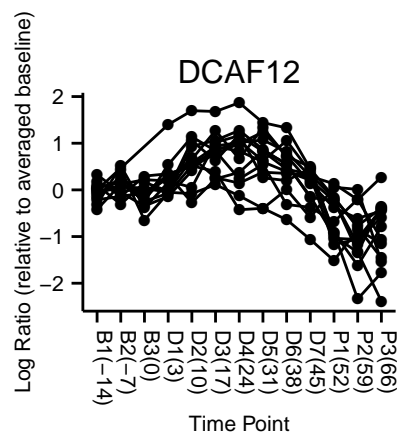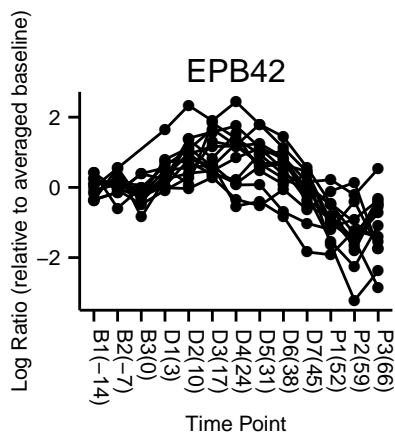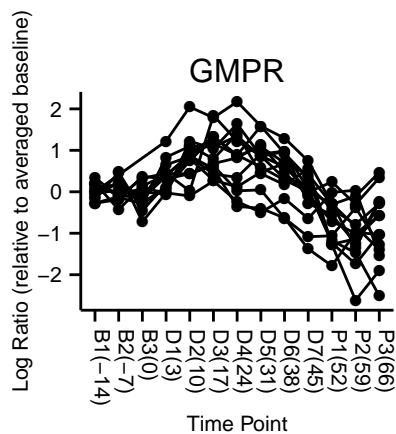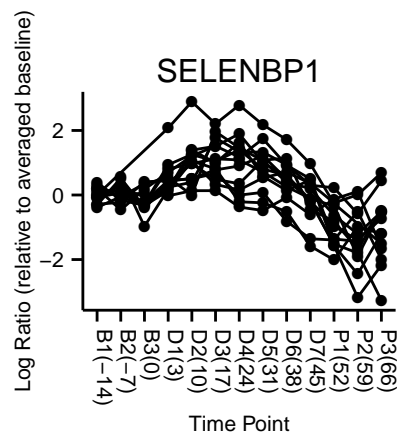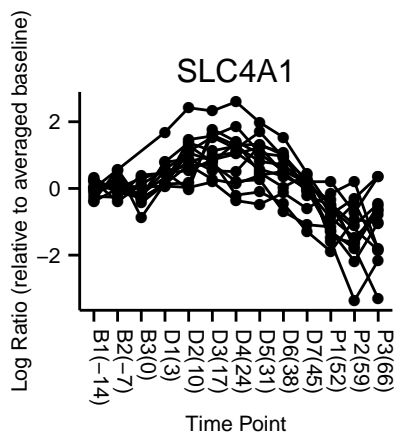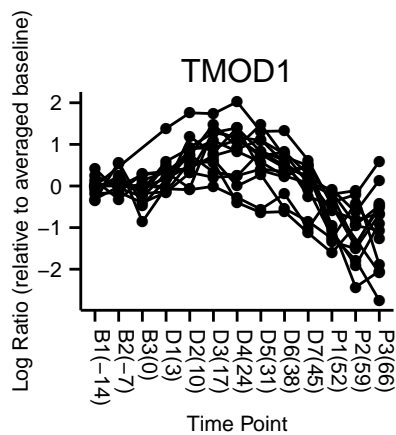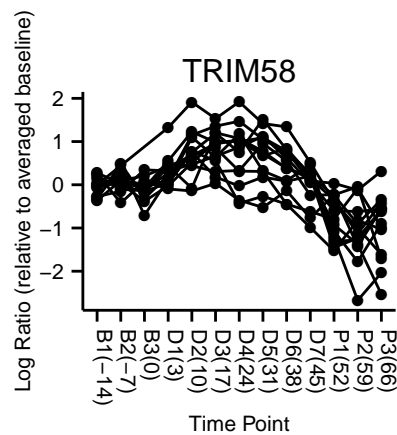

Supplement: Supplementary file 5 — Individual expression of the ALAS2, BCL2L1, DCAF12, EPB42, GMPR, SELENBP1, SLC4A1, TMOD1 and TRIM58 genes over time in response to rHuEpo in the MDS, respectively. (PDF 23 kb) [file 12864_2017_4191_MOESM5_ESM.pdf]

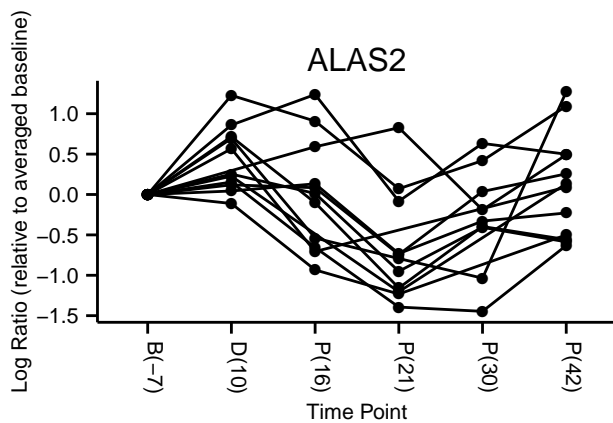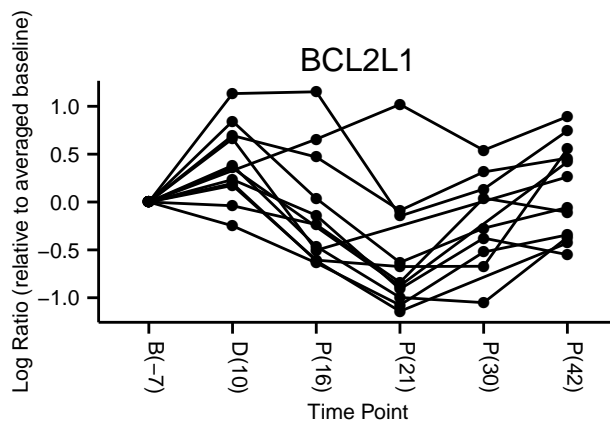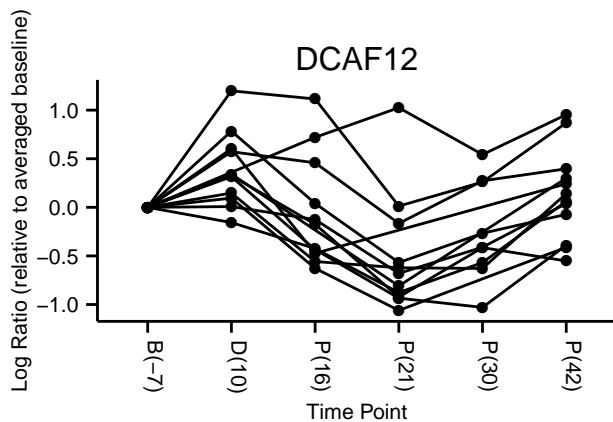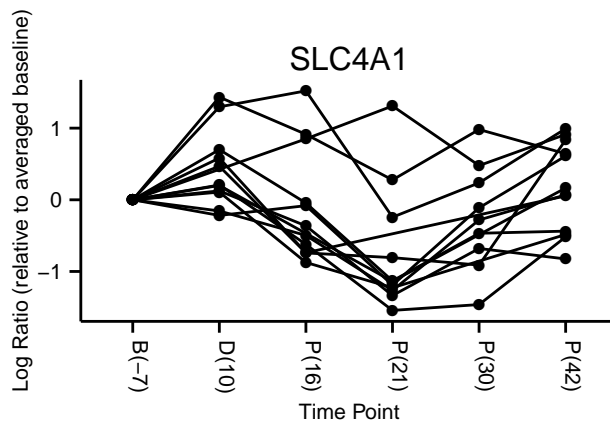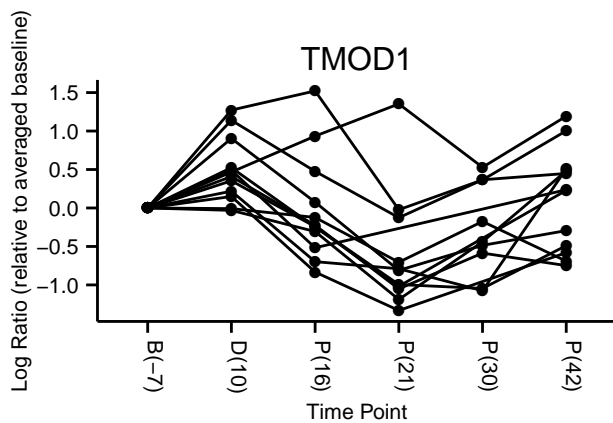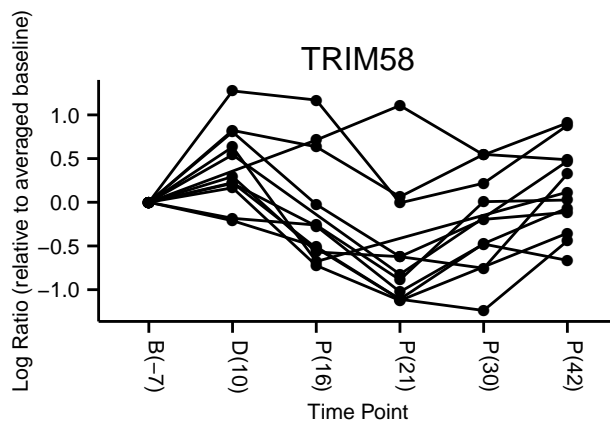

Supplement: Supplementary file 8 — Individual expression of the ALAS2, BCL2L1, DCAF12, SLC4A1, TMOD1 and TRIM58 genes over time in response to altitude exposure in the 12 ATS elite runners, respectively. B(−7), D(10), P(16), P(21), P(30) and P(42) represent pre, during, 48-h-, 1-week-, 2-week- and 4-week-post altitude exposure, respectively. (PDF 9 kb) [file 12864_2017_4191_MOESM8_ESM.pdf]
